# Cyanoacrylate Injection Versus Band Ligation in the Endoscopic Management of Acute Gastric Variceal Bleeding: Meta-Analysis of Randomized, Controlled Studies Based on the PRISMA Statement

**DOI:** 10.1097/MD.0000000000001725

**Published:** 2015-10-16

**Authors:** Weiguang Qiao, Yutang Ren, Yang Bai, Side Liu, Qiang Zhang, Fachao Zhi

**Affiliations:** From the Guangdong Provincial Key Laboratory of Gastroenterology (WQ, YB, SL, QZ, FZ), and Department of Gastroenterology, Nanfang Hospital, Southern Medical University, Guangzhou, China (WQ, YB, SL, QZ, FZ); and Department of Gastroenterology, Beijing Tsinghua Changgung Hospital, Medical Center, Tsinghua University, Beijing, China (YR).

## Abstract

The evidence for optimal endoscopic management of bleeding gastric varices is lacking. The clinical outcome is controversial in trials comparing cyanoacrylate injection and band ligation. To help guide endoscopic decisions regarding acute gastric variceal bleeding, a meta-analysis was conducted.

Cochrane Central Register of Controlled Trials, MEDLINE, EMBASE, and ScienceDirect were searched for all randomized controlled trials (RCTs) containing the 2 interventions. The main outcomes evaluated in the meta-analysis were active bleeding control, blood transfusion, rebleeding, recurrence of varices, complications, and survival.

Three RCTs were identified, which included 194 patients with active gastric variceal bleeding from Taiwan and Romania. Active bleeding control was achieved in 46 of 49 (93.9%) patients in the cyanoacrylate injection group, compared with 35 of 44 (79.5%) in the band ligation group (*P* = 0.032), for a pooled odds ratio of 4.44 (95% confidence interval, 1.14–17.30). Rebleeding rate was comparable in type 2 gastroesophageal varices (GOV2) between the 2 interventions (35.7% vs 34.8%, *P* = 0.895), but cyanoacrylate injection seemed superior for reducing rebleeding rate in type 1 gastroesophageal varices (GOV1, 26.1% vs 47.7%, *P* = 0.035) and type 1 isolated gastric varices (IGV1, 17.6% vs 85.7%, *P* = 0.015). Cyanoacrylate injection was also superior in controlling recurrence of gastric varices to band ligation (36.0% vs 66.0%, *P* = 0.002). There was no difference in complications or mortality between the 2 interventions. The major limitation of this meta-analysis is the small number of studies/patients included.

Compared with band ligation, injection cyanocrylate have an advantage in the control of acute gastric variceal bleeding, also with lower recurrence rate and rebleeding (except GOV2). The limited amount of studies included attenuates the strength of this meta-analysis; therefore, more high-quality RCTs are needed.

## Abstract 2

Supplemental Digital Content is available in the text

## INTRODUCTION

Gastroesophageal varices (GOV), containing esophageal varices (EV) and gastric varices (GV), are the common complications of portal hypertension, which may result in massive gastrointestinal hemorrhage. The American Society of Gastrointestinal Endoscopy (ASGE) guidelines,^1^ the National Institute for Health and Clinical Excellence (NICE) guideline,^2^ and the American Association for the Study of Liver Diseases (AASLD) practice guideline^3^ recommend endoscopic hemostasis as the first-line management after initial resuscitation with fluids and packed red blood cells. Although acute GV bleeding is not as prevalent as EV bleeding, it is more severe with higher mortality and treatment failure (>30%).^4^ Over the past years, the endoscopic treatment for EV bleeding has made profound progress. However, high-quality data for the endoscopic therapy of acute GV bleeding are lacking. Current available endoscopic options include sclerotherapy, band ligation, and cyanoacrylate injection.^5^ Finding the best endoscopic management is of paramount importance and could be life saving.

Furthermore, Sarin et al^6^ categorized GV into gastroesophageal varices (GOV) and isolated gastric varices (IGV). Type 1 GOVs (GOV1) extend below the gastroesophageal junction along the lesser curvature of the stomach. Type 2 GOVs (GOV2) extend below the gastroesophageal junction into the fundus. Type 1 IGVs (IGV1) are only located in the fundus and type 2 IGVs (IGV2) are located elsewhere in the stomach. This classification of gastric varices could have different bleeding risk and treatment failure (eg, IGV1s have the highest bleeding incidence and the highest rebleeding rate), which would have important clinical implications regarding the best choice of endoscopic management. However, the evidence is limited.

Sclerotherapy with polidocanol or pure alcohol was first introduced for obliterating varicose veins with great success in acute EV bleeding. But it was abandoned for the management of acute GV bleeding because of high rebleeding rates (50% to 90%).^5^ Band ligation is recommended by the ASGE and Baveno V consensus for acute EV bleeding.^1,7^ For acute GV bleeding, it may also be used for those patients with GOV1 and small GOV2. However, it is not suggested for large (>2 cm) GOV2 or IGV1.^5^ Cyanoacrylate injection, the most popular tissue glue of the world,^8^ is also recommended for acute GV bleeding with a high rate of bleeding control (>90%). It is the therapy of choice for GOV1, GOV2, and IGV1 in experts’ opinion.^7^ However, in rural hospitals where cyanoacrylate is not available, band ligation may be the therapy of choice for its “easy to learn.”^9^ It has proven safe and effective for cessation of acute GV bleeding by ligation.^10,11^

There are only a few small-size randomized controlled studies (RCTs) comparing the effectiveness of cyanoacrylate injection and band ligation in acute GV bleeding with conflicting results. Lo et al^12^ conducted the first randomized controlled study in 2001 and found that cyanoacrylate injection was more effective than band ligation for acute bleeding control. Later in 2006, Tan et al^13^ reported equal results for arresting acute GV bleeding (93.3% vs 93.3%, respectively). More recently, Tantao et al^14^ suggested comparable bleeding control rates (100% vs 88.9%, *P* = 0.43). Nevertheless, there are few studies that evaluated the clinical outcome of GV subtypes according Sarin's classification. The optimal management of acute GV bleeding remains controversial.

The purpose of this study was to compare, by using meta-analysis, the effectiveness of cyanoacrylate injection vs band ligation for patients with acute GV bleeding. The outcomes of interest were acute bleeding control, therapeutic sessions, GV eradication and recurrence, rebleeding rate, complications, and mortality.

## MATERIALS AND METHODS

The PRISMA (Preferred Reporting Items for Systematic Reviews and Meta-Analyses) statement and guidelines were consulted during the stages of design, analysis, and reporting of this meta-analysis.^15,16^ This is a meta-analysis, therefore an ethics committee and/or institutional board approval was not required. This systematic review protocol was registered within the International Prospective Register of Systematic Reviews (PROSPERO) as number CRD42013005286 and is available in full on the NIHR (National Institute for Health Research) website.

### Study Identification and Selection

We searched medical literature by using MEDLINE (between October 1978 and November 2014), ScienceDirect (between April 1981 and November 2014), EMBASE, and Cochrane Central Register of Controlled Trials. Subject headings and keywords for gastric variceal bleeding or hemorrhage were combined with ligation and text word variants for “cyanoacrylate” or “tissue glue.” We included human clinical trials published in any language. In addition, a manual search was performed with references from retrieved reports, review articles, editorials, and textbooks of gastroenterology. This process was performed by 2 authors (WQ and YR) with the assistance of a university medical librarian.

Studies were selected if they met the following inclusion criteria: study design as RCT; study population was patients with acute gastric variceal bleeding; interventions were endoscopic with cyanoacrylate injection compared with band ligation; and 1 or more of following outcomes: active bleeding control, blood transfusion, therapeutic sessions, GV eradication and recurrence, rebleeding rate, complications, and mortality. We excluded studies that are: cohort study or case–control study; study without extractable data; published as a case report, editorials, reviews, and letters to the editor.

### Assessment of Bias

Two investigators (WQ and YR) independently evaluated the selected studies using the Cochrane Collaboration's tool for assessing risk of bias of the randomized controlled studies. The following aspects were included: sequence generation, allocation concealment, blinding of participants, personnel and outcomes assessors, description of the completeness of outcomes data for each main outcome, assessment of selective reporting, and other sources of bias specific to the study. Each factor will be rated as “low risk,” “high risk,” and “unclear risk.” Disagreements were resolved by discussion.

### Outcomes

The primary outcome of interest was active bleeding control as evaluated endoscopically. The secondary outcomes were units of blood transfusion, treatment sessions of the 2 interventions, GV eradication and recurrence, rebleeding rate, complications, and mortality. GV eradication was defined as nonvisualization of any patent GV. Recurrence refers to visualization of GV after confirmed eradication. Complications were defined as any untoward events (eg, ulcer bleeding from gastric varices, bacteremia, or bacterial peritonitis) that required treatment or prolonged hospitalization. Mortality means patients died during treatment or hospitalization.

### Data Extraction

Two investigators (WQ and YR) independently extracted the available data (acute bleeding control, treatment sessions, GV eradication and recurrence, rebleeding rate, complications, and mortality) to determine whether these trials could be combined. Discrepancies were resolved by discussion. The following data were also collected for each included study: first author of study, year(s) conducted/published, country and geographical region, study duration, number of patients, and follow-up period.

### Data Analysis

The effectiveness of cyanoacrylate injection versus band ligation was calculated using a pooled estimate of odds ratio (OR) with 95% confidence intervals (CIs). α = 0.05 was set as the statistical significance level. Heterogeneity was calculated using the χ^2^ test and I^2^ statistic. Heterogeneity was considered significant if the *P* values were ≤0.1 and I^2^ was ≥50%. The fixed-effect model was employed if there was no evidence of heterogeneity; otherwise, the random-effect model was used. For treatment sessions particularly, the standard mean difference (SMD) and 95% CIs were calculated based on random-effect model. Publication bias was not assessed because of the low power to detect a difference between chance and true asymmetry when <10 studies are included. Subgroup analysis was carried out for the rebleeding rate according to Sarin's classification of GV.^6^ STATA version 12.0 (StataCorp, College Station, TX) was used for statistical analysis.

## RESULTS

### Descriptive and Qualitative Assessment

**FIGURE 1:**
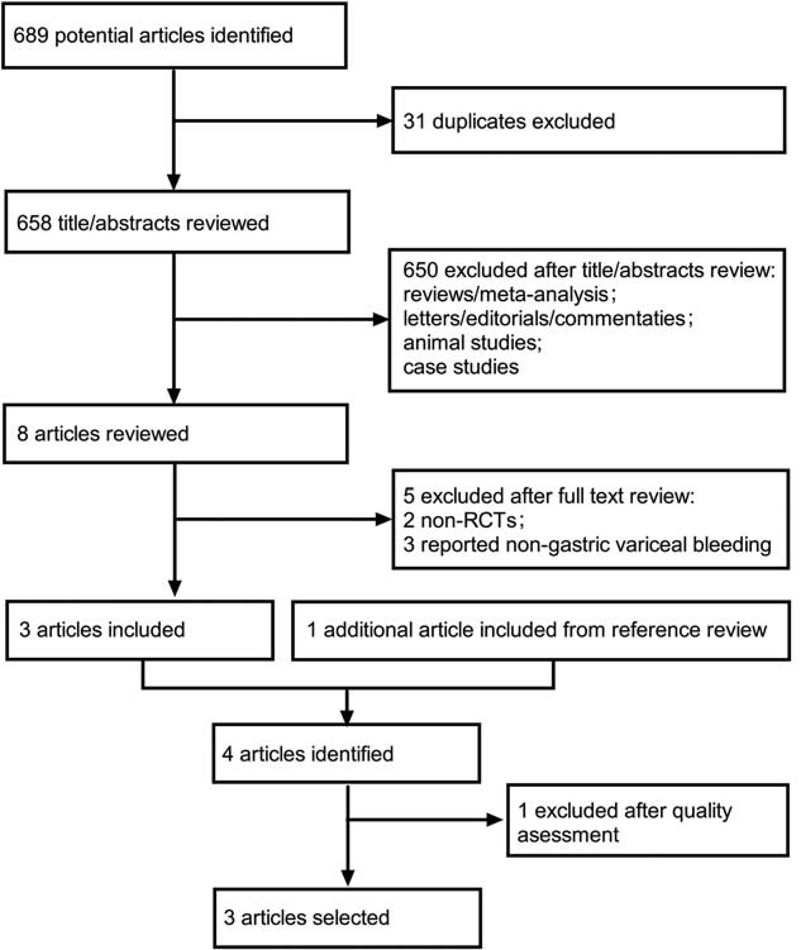
Meta-analysis flow diagram of study selection. RCT = randomized control trial.

**FIGURE 2:**
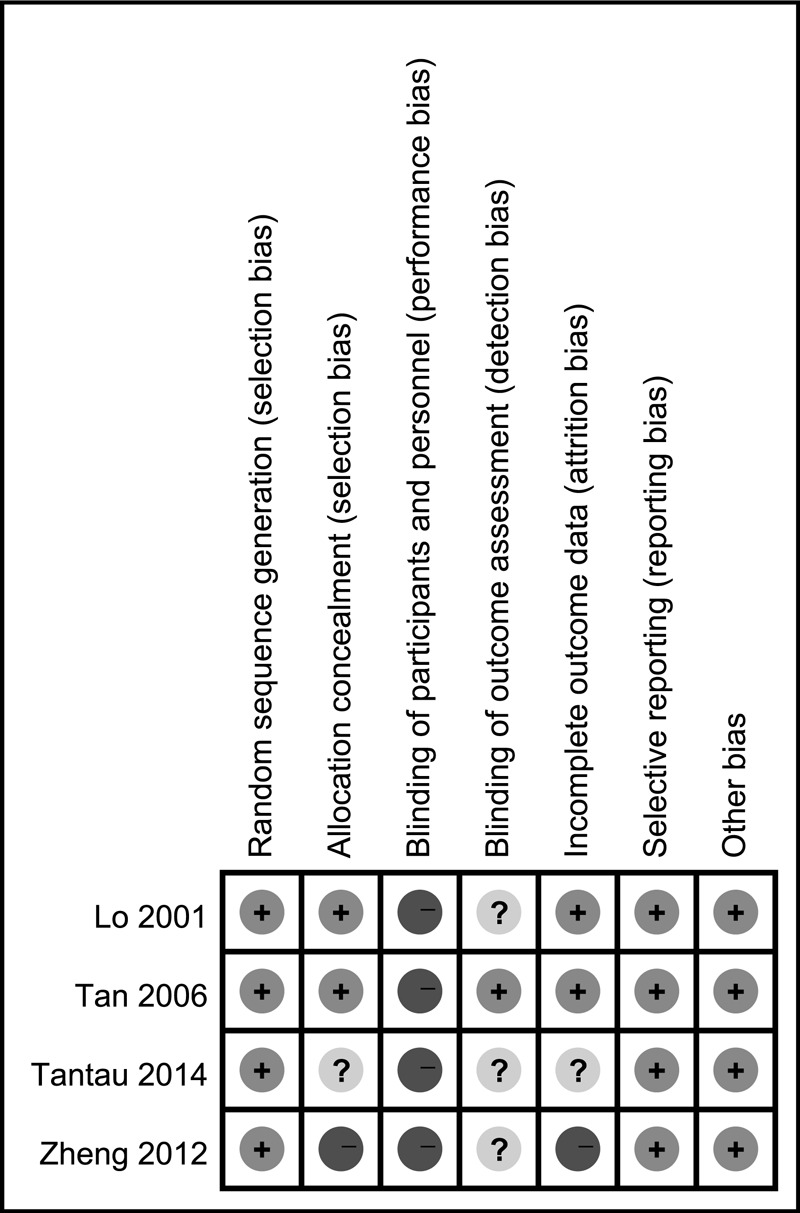
Risk of bias summary: authors’ judgments about each risk of bias item for each included study. Green circles with +, low risk of bias; Yellow circles with ?, unclear risk of bias; Red circles with −, high risk of bias.

A total of 689 potential references were identified after searching, and 686 articles were excluded after abstract or full text review. Figure 1 summarizes the meta-analysis flow. One additional article was included from the reference review, but was excluded concerning the methodological quality.^17^ None of the 3 remaining RCTs (Lo et al^12^, Tan et al^13^, Tantau et al^14^) scored high risk of bias on study or outcome level (Fig. 2) and were included for meta-analysis.^12–14^ Methodological characteristics of all 3 trials are shown in Supplementary Table, http://links.lww.com/MD/A459. The 3 trials were full-length articles of comparable quality, which enrolled patients with acute GV bleeding, managed with cyanoacrylate injection or band ligation. The included patients also received medical therapy, which included general supportive care, vasoactive drugs (eg, terlipressin or somatostatin analogs), blood transfusion, and systemic antibiotics. One trial used beta-blockers as additional secondary prophylaxis (Tantau et al^14^).

Table 1 summarizes the outcome data of the 3 RCTs. The total number of subjects was 194; 99 were randomized to the cyanoacrylate injection group and 95 to the band ligation group. One trial found cyanoacrylate injection was superior to band ligation for active bleeding control, and 2 other trials found no difference. In the 2 studies in which the rebleeding rate of GV subtypes was analyzed, there were controversial results on prevention of IGV1 rebleeding.

**TABLE 1:**
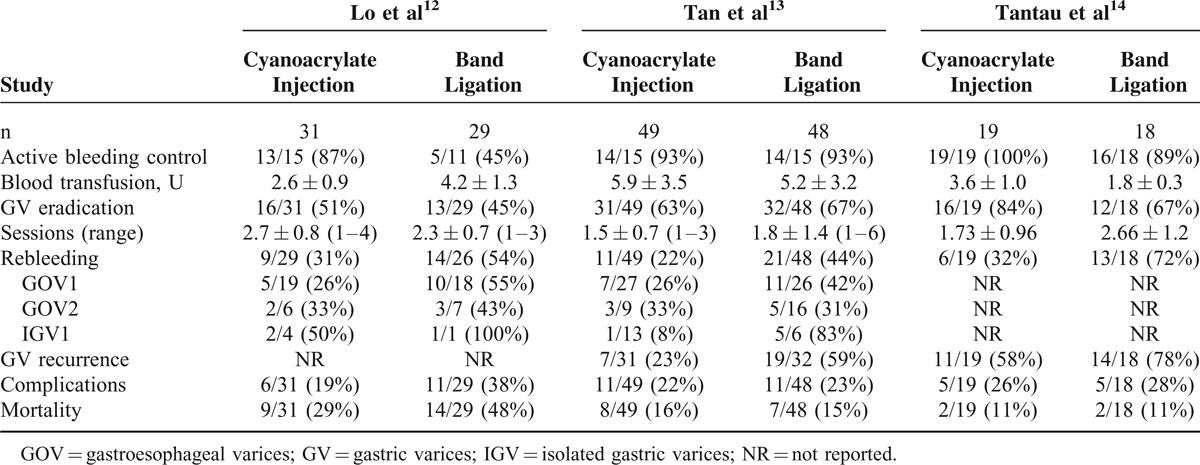
Clinical Outcome of the Included Trials

### Quantitative Assessment of Clinical Outcomes

Table 2 summarized the comparisons of several clinical outcomes: active bleeding control, blood transfusion, GV eradication, treatment sessions, rebleeding rate, complications, GV recurrence, and mortality in the meta-analysis for cyanoacrylate injection versus band ligation.

**TABLE 2:**
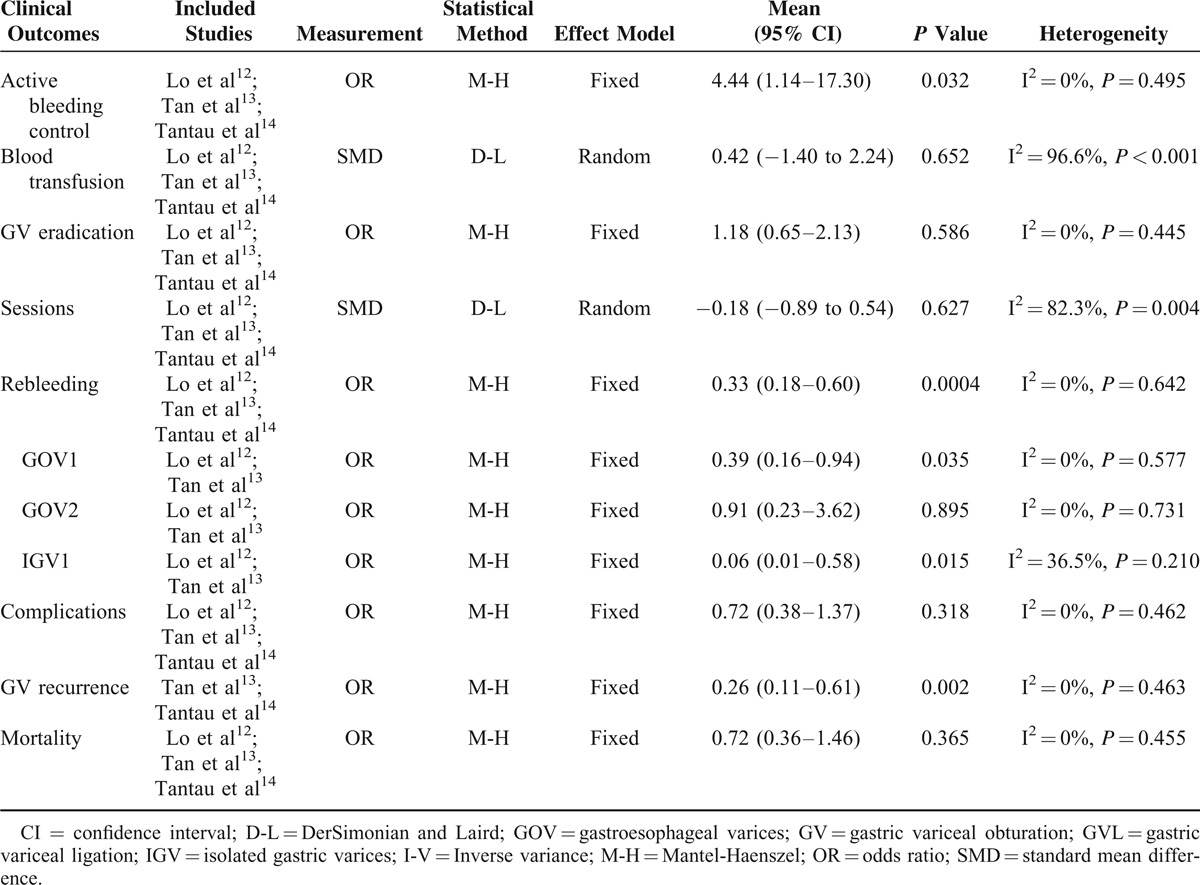
Meta-Analysis of Comparison Between Cyanoacrylate Injection and Band Ligation in Acute Gastric Variceal Bleeding

**FIGURE 3:**
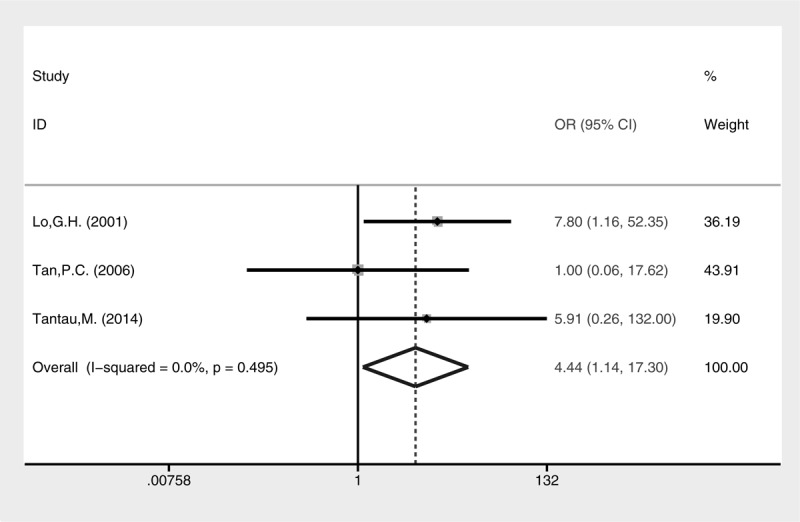
Meta-analysis of arresting active GV bleeding in groups of cyanoacrylate injection and band ligation.

**FIGURE 4:**
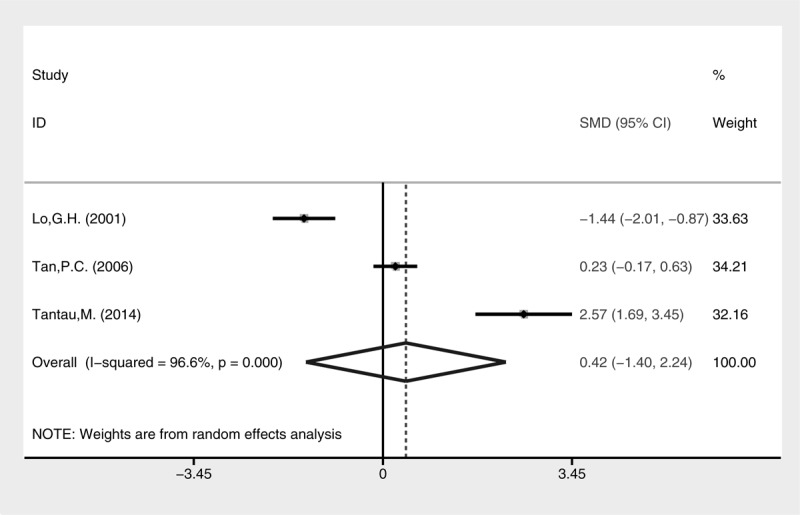
Meta-analysis of blood transfusion in groups of cyanoacrylate injection and band ligation.

**FIGURE 5:**
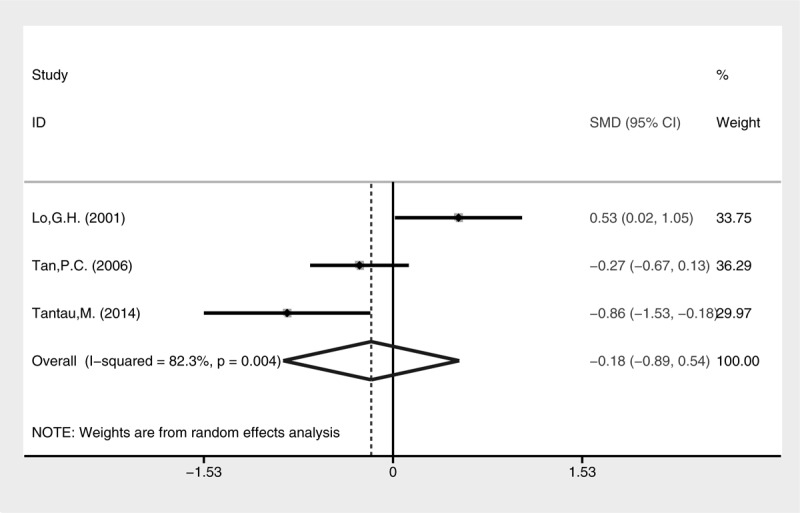
Meta-analysis of treatment sessions in groups of cyanoacrylate injection and band ligation.

**FIGURE 6:**
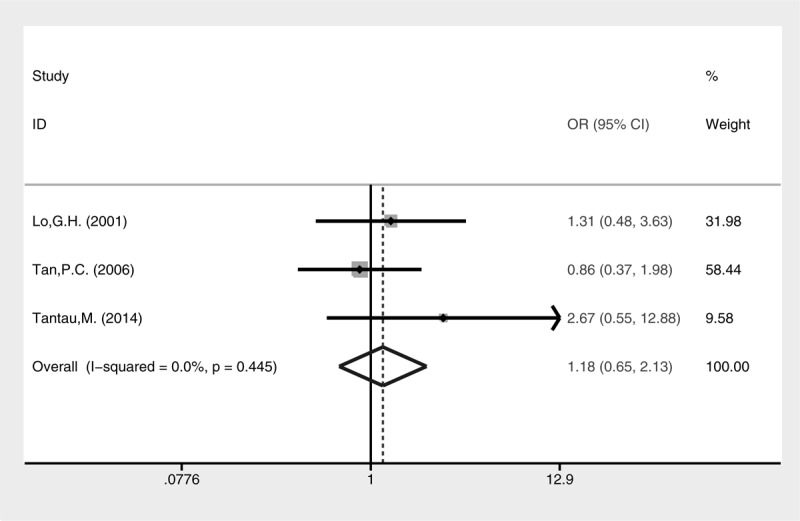
Meta-analysis of GV eradication in groups of cyanoacrylate injection and band ligation.

**FIGURE 7:**
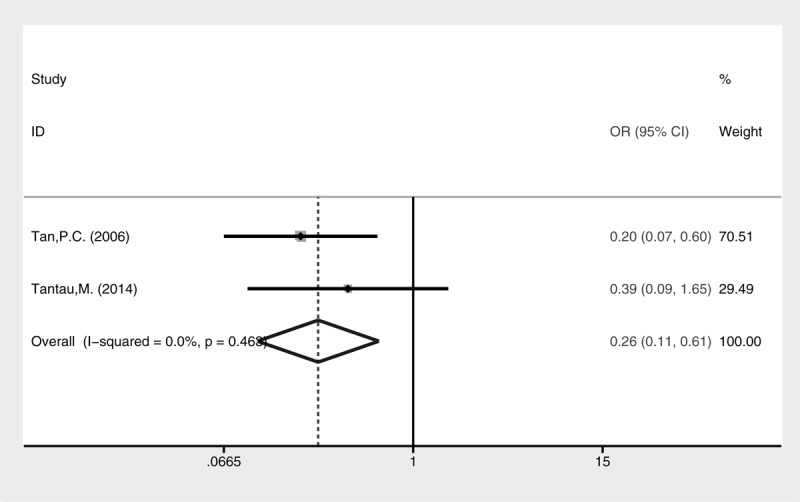
Meta-analysis of GV recurrence in groups of cyanoacrylate injection and band ligation.

Higher rate of active bleeding control was revealed in the cyanoacrylate injection group (Fig. 3, fixed-effect model, OR = 4.44, 95% CI = 1.14–17.30, *P* = 0.032). No statistical difference of blood transfusion was detected between the 2 groups (Fig. 4, random-effect model, SMD = −0.42, 95% CI = −1.40 to 2.24, *P* = 0.652), although heterogeneity was present (I^2^ = 96.6%, *P* < 0.001). The treatment sessions until the varices were obliterated were also comparable between the 2 groups (Fig. 5, random-effect model, SMD = −0.18, 95% CI = −0.89 to 0.54, *P* = 0.627). The rate of GV eradication reveals no statistic difference (Fig. 6, fixed-effect model, OR = 1.18, 95% CI = 0.65–2.13, *P* = 0.586). Furthermore, lower GV recurrence rate was revealed in cyanoacrylate injection group (Fig. 7, fixed-effect model, OR = 0.26, 95% CI = 0.11–0.61, *P* = 0.002).

**FIGURE 8:**
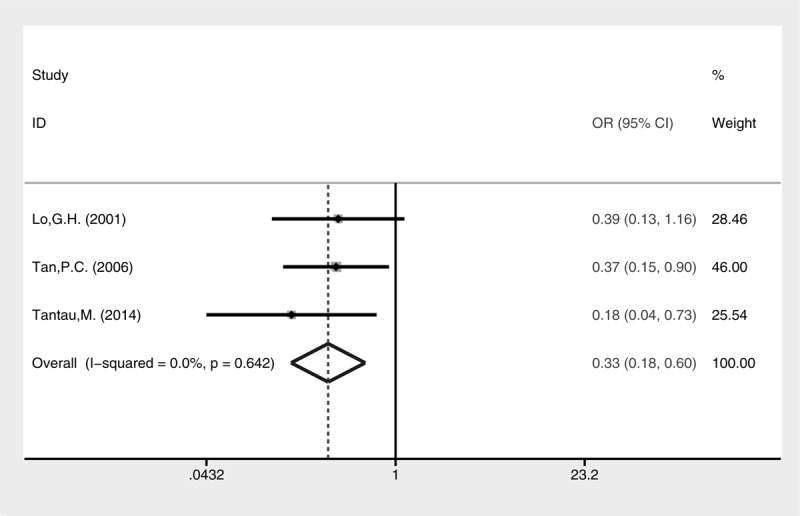
Meta-analysis of GV rebleeding in groups of cyanoacrylate injection and band ligation.

**FIGURE 9:**
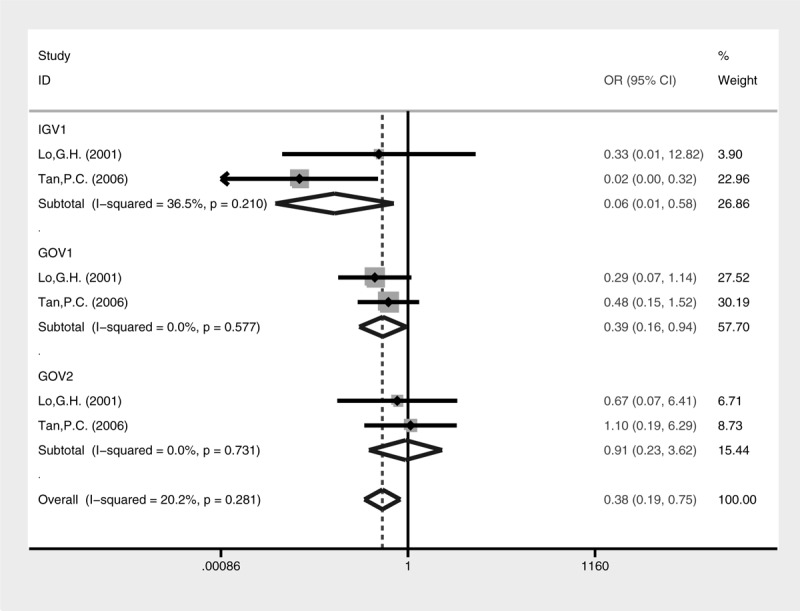
Meta-analysis of rebleeding in GV subtypes treated with cyanoacrylate injection and band ligation.

Lower rebleeding rate was also revealed in the cyanoacrylate group (Fig. 8, fixed-effect model, OR = 0.33, 95% CI = 0.18–0.60, *P* = 0.0004). A subgroup analysis of different types of GV for rebleeding was conducted (Fig. 9). Cyanoacrylate injection was better for prophylaxis of GV rebleeding in IGV1 (fixed-effect model, OR = 0.06, 95% CI = 0.01–0.58, *P* = 0.015) and GOV1 (fixed-effect model, OR = 0.39, 95% CI = 0.16–0.94, *P* = 0.035). However, this advantage was not observed in GOV2 (fixed-effect model, OR = 0.91, 95% CI = 0.23–3.62, *P* = 0.895).

**FIGURE 10:**
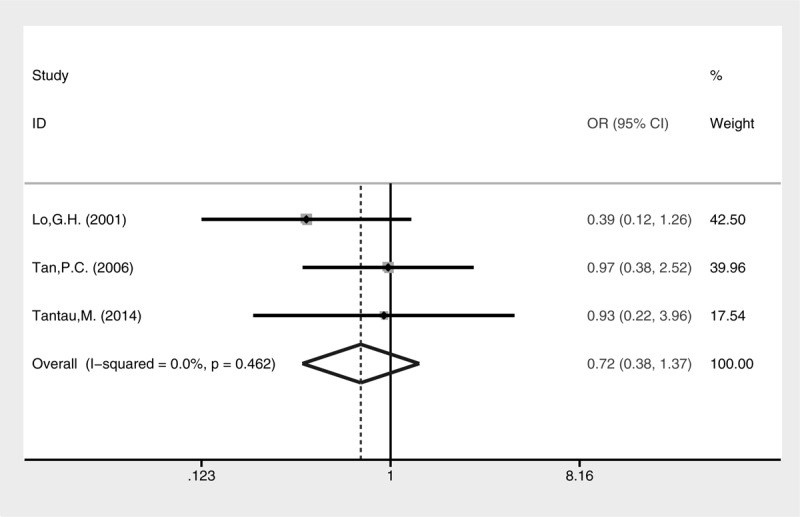
Meta-analysis of complications in groups treated with cyanoacrylate injection and band ligation.

**FIGURE 11:**
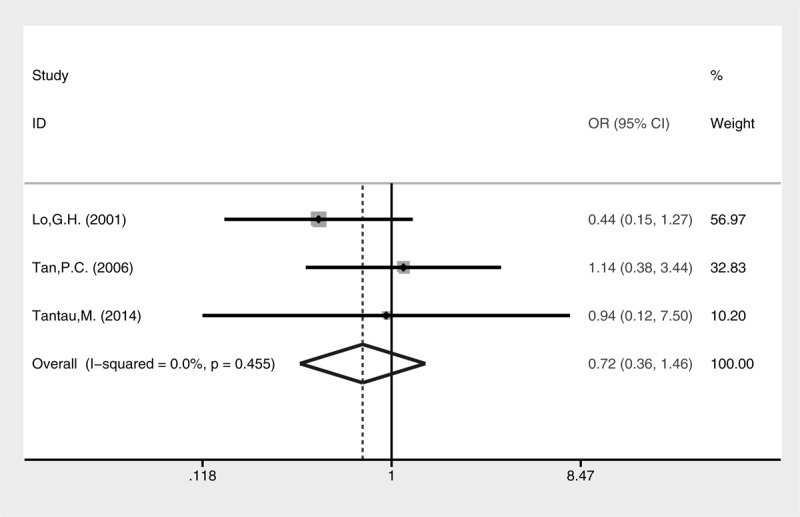
Meta-analysis of mortality in groups treated with cyanoacrylate injection and band ligation.

Both complication rate (Fig. 10, fixed-effect model, OR = 0.72, 95% CI = 0.38–1.37, *P* = 0.318) and mortality (Fig. 11, fixed-effect model, OR = 0.72, 95% CI = 0.36–1.46, *P* = 0.365) were comparable between cyanoacrylate injection and band ligation.

## DISCUSSION

Identification of appropriate endoscopic treatment for acute GV bleeding can help guide clinical decision-making regarding hemostasis efficiency and survival benefit. Cyanoacrylate injection was suggested for GV bleeding by ASGE guidelines, based on low-quality evidence.^1^ High-quality randomized clinical trials with other endoscopic therapies such as band ligation are lacking, yielding controversial results. This meta-analysis examined 3 high-quality RCTs comparing endoscopic cyanoacrylate injection and band ligation for acute GV bleeding. The results suggested that cyanoacrylate injection is superior to band ligation in acute bleeding control, variceal recurrence, and prophylaxis for rebleeding, especially in IGV1 and GOV1 subtypes.

Although GV is not as frequent as EV, bleeding from GV is usually more severe and even more fatal than from EV. Because GV lies deeper, on average are larger, drain directly into large veins without intervening smaller veins, and are exposed to acid and pepsin. Endoscopic therapy for the treatment of bleeding GV is less successful than for EV.^18^ Current recommended endoscopic interventions are cyanoacrylate injection and band ligation. However, there is no general consensus regarding the optimal management strategy for this condition.

Cyanoacrylate injection was originally reported with rapid control of variceal bleeding by Soehendra et al.^19^ It has been suggested for the treatment of GV bleeding by ASGE guideline,^1^ Baveno consensus,^7^ NICE guideline,^2^ as well as AASLD practice guideline.^3^ In observational studies, Rengstorff and Binmoeller^20^ reported that injection with cyanoacrylate achieved an immediate hemostasis rate with 100%, only 4% of patients encountered bleeding recurred by a mean follow-up 11 months. Similar results were found by Mumtaz et al^21^ with a 100% primary hemostasis and 14% re-bleeding rate. Marques et al^22^ also reported a primary hemostasis was accomplished in 42 patients (87.5%).

On the contrast, band ligation was recommended to arrest EV bleeding by ASGE guideline,^1^ Baveno consensus,^5^ NICE guideline,^2^ as well as AASLD practice guideline.^3^ If cyanoacrylate is not available such as in rural hospitals, band ligation could be considered an alternative.^23^ Ligation is often chosen by endoscopists in the emergency unit for its “easy to learn,” which proved to be safer and equally efficacious than sclerosing agents.^9^ It has proven safe and effective for cessation of acute GV bleeding (83% to 100%).^10^ GV eradication by ligation could be achieved in 91% patients.^23^ Traditionally, band ligation is not recommended for large (>2 cm) bleeding GV. However, Lee et al^24^ reported a hemostatic rate of 82.9% for treatment of large bleeding GV using large detachable snares.

Better arresting active bleeding is of paramount importance. Current available RCTs yielded inconsistent results. Lo's study shows that cyanoacrylate injection was superior to band ligation for arresting active bleeding, while in Tan's and Tantau's study, both treatments are comparable. It is undeniable that small sample size might not have enough power to detect statistical significance. But there might be other confounding factors like differences in the endoscopists’ personal experience with cyanoacrylate injection and band ligation. It is noteworthy that in Lo's study, large (F3) gastric varices were included. For general practice, endoscopic band ligation could be used for small (<2 cm) gastric varices as an alternative, probably because of limited diameter of the standard ligator.^5^ This possibly explains lower efficacy in arresting active bleeding with band ligation.

Another key element is the rebleeding rate after endoscopic management, because of increased mortality during the rebleeding episode. There was no controversy in the results of the included RCTs. This meta-analysis suggested that rebleeding rate the band ligation group was 3 times higher than that in the cyanoacrylate injection group. The difference in the efficacy of hemostasis between the 2 endoscopic therapies may be attributed as follows: the movement of the stomach might slough the ligated rubber band whereas it would have less impact on the injected varices; the ligation would have effect mainly on the superficial collaterals in the mucosa and submucosa, while the cyanoacrylate could obliterate collaterals over a wider area and in deeper layers; different hemostatic mechanism: cyanoacrylate polymerizes rapidly and plugs the lumen if injected into varices, in contrast, ligation results in strangulation and necrosis of varices. There is no doubt that the necrosis of varices caused by ligation is slower than the embolism caused by cyanoacrylate.

More importantly, a subgroup analysis of rebleeding rate has been conducted according to Sarin's classification, which might have impact on clinical decision. In Lo's study, the rebleeding rate was comparable between the 2 interventions regardless of GOV1, GOV2, or IGV1 subgroups. In Tan's study, similar results were found in GOV1 and GOV2, except that cyanoacrylate injection was superior to band ligation for preventing rebleeding in IGV1. Based on the meta-analysis, the cyanoacrylate injection could be a better choice for prophylaxis of rebleeding in IGV1. However, it was unexpected that the former choice was still superior in GOV1. It has been suggested that EV and GOV1 have different blood drainage from GOV2 and IGV1. The portal blood flow is reversed through the right and left gastric veins around the distal esophagus and cardia (EV and GOV1), while it is through the short and posterior gastric vein around gastric fundus (GOV2 and IGV1).^25^ The result is unexplainable on the aspect of pathophysiology. The Baveno V consensus workshop suggested that GOV1 bleeding could be equally treated with band ligation as EV bleeding, but recommended the use of cyanoacrylate injection as a better option for GOV2 and IGV1 bleeding.^7^ On the other hand, some experts questioned that GOV1 should not be treated equally as for EV.^26,27^ Our meta-analysis supported the later perspective that cyanoacrylate injection is also the preferred option for GOV1 bleeding.

Although the total complication rate between cyanoacrylate injection and band ligation was comparable in the meta-analysis, what we care is the postprocedural ulcer or ulcer bleeding. This complication was only stated in Lo's study, making the subgroup analysis impossible. The huge ulcer on GV was comparable after the 2 interventions, but band ligation had significantly more bleeding from ulcers on GV. The safety and efficacy of treating GV bleeding with band ligation was also questioned by Lo et al, which also supported our findings.^16^

This meta-analysis has some blemish. The major limitation of this meta-analysis is the small number of studies/patients included. Only 3 studies were included, with a total of only 194 patients (<100 for each treatment). The limited amount of evidence included attenuates the strength of this meta-analysis. Thus, more studies are needed to interpret this question. Besides, there is clinical heterogeneity with variations in patient comorbidity, the rate and amount of variceal bleeding, supportive care, and endoscopists’ experience (eg, in Lo's study, a single ligator was applied instead of multiple ligators used in other studies). Heterogeneity could also be detected in comparing blood transfusion and treatment sessions, but it is minimal in most other comparisons, such as active bleeding control and GV rebleeding. Finally, control of active bleeding in GV subtypes was only analyzed in Lo's study, which showed equal outcome in GOV2 but insignificant higher efficacy in GOV1 with cyanoacrylate injection. So the outcome of the endoscopic interventions for GV subtypes could not be analyzed except for rebleeding. Besides, there is no comparative study on IGV2. Based on the above limitations, the results of this meta-analysis should be interpreted with caution. More high-quality RCTs are still needed to propound more evidence on treatment decisions.

In conclusion, compared with band ligation, injection cyanocrylate have an advantage in the control of acute gastric variceal bleeding, also with lower recurrence rate and rebleeding (except GOV2). The 2 interventions seem equivalent with regard to their effect on transfusion requirement, treatment sessions, complications, and mortality. The limited amount of studies included attenuates the strength of this meta-analysis; therefore, more high-quality RCTs are needed.
